# Introduction of a Third Trimester Pregnancy Patient Education Guide and Effects on Patient Satisfaction With Counseling: A Prospective Cohort Study

**DOI:** 10.1111/jep.70202

**Published:** 2025-07-06

**Authors:** Clarice Hu, Julie Hurvitz, Jessica K. Lee

**Affiliations:** ^1^ School of Medicine University of Maryland Baltimore Maryland USA; ^2^ Department of Obstetrics, Gynecology and Reproductive Sciences University of Maryland Baltimore Maryland USA

**Keywords:** educational guide, patient education, patient satisfaction, prenatal care

## Abstract

**Aims and Objectives:**

To assess whether a workflow change introducing a third trimester pregnancy education guide would be associated with increased patient satisfaction regarding prenatal counseling.

**Methods:**

We surveyed pregnant patients between 34w0d‐41w6d during a single prenatal appointment at one of two hospital‐associated offices. We enrolled 49 ‘pre‐guide’ patients March‐May 2023 and 50 ‘post‐guide’ patients October–December 2023, after a 4‐month washout period. We performed *t*‐tests and chi‐squared analyses to compare the groups.

**Results:**

The cohorts had similar sociodemographic characteristics aside from lower education level in the post‐guide cohort (*p* = 0.01). There was no significant difference between the groups and their reported satisfaction with third trimester and delivery counseling (*p* = 0.92). Those who received the guide were more likely to feel they were more adequately counseled regarding ‘pain management in childbirth’ (*p* = 0.01), but there were no other statistically significant differences between the groups. Of those who received the guide, 75% felt that it reduced their anxiety and stress about the unknown and 100% would recommend it to others.

**Conclusions:**

We did not find that introduction of an informational guide about the third trimester of pregnancy significantly affected their satisfaction with their third trimester and delivery counseling or patient perception of the adequacy of counseling on specific pregnancy topics. All patients who received the guide noted they would recommend it to others.

## 1 Introduction

In an increasingly digital and connected age, patients have unprecedented access to written medical information through websites and apps that may not be created by healthcare professionals [1]. Patients express that they value advice from experts the most [1, 2] and desire more information from health care professionals [1, 3]. Furthermore, patients have been shown to lack crucial knowledge with regard to pregnancy such as warning signs of abnormal fetal movement [4] and they also reported not feeling prepared for childbirth and the postpartum period [5], suggesting the need for more counseling from providers. This lack of preparation for childbirth and the postpartum period was associated with lower satisfaction with their clinicians [5].

Though patients prefer face‐to‐face education first and foremost [2, 3], educational materials have been received favorably by patients and provided benefits to patients as well. In one study, digitized weekly pregnancy guide improved patient satisfaction and increased loyalty to the healthcare team [6]. Furthermore, patients were overall satisfied with the educational pamphlets themselves [7, 8]. Patient mental health is another important factor that can be improved by written materials. When researchers implemented an educational pamphlet for patients with pre‐eclampsia, patients who received the pamphlet expressed lowered anxiety levels [7]. Furthermore, decision aids/informational pamphlets help patients feel more empowered and informed when making decisions about their pregnancies [1, 8, 9].

As part of a quality improvement initiative, our institution released a new patient‐centered educational pamphlet for the third trimester of pregnancy. This study aims to understand the effects of the release of this guide on patients, especially as there have been limited to no studies on the effects of a clinician‐written guide about the third trimester of pregnancy. Primarily, we hypothesized that this guide would improve patient satisfaction in prenatal education and care. Secondary outcomes included effects on patient reported anxiety, satisfaction with the guide itself, and adequacy of counseling regarding topics of pregnancy and pregnancy care.

## 2 Methods

We performed a prospective cohort study analyzing the effects of an educational guide on patient knowledge and satisfaction. This study was determined to be exempt by the University of Maryland Institutional Review Board. The guide provided additional guidance on the third trimester of pregnancy including information about laboratory tests in the third trimester of pregnancy, preparation for delivery, contraceptive choices, and pain management during labor. We based sample size calculations on 90% power (and 95% confidence) to detect a 1‐point (or 20%) increase in patient satisfaction rate with third trimester and delivery counseling (on Likert scale 1−5) between groups surveyed. This required enrolling at least 21 patients per group for a total sample size of 42.

One member of the research team consented and administered anonymous surveys to English‐speaking pregnant patients between 34w0d‐41w6d during a single prenatal appointment at one of two hospital‐associated outpatient offices. We conducted surveys of the first group, ‘pre‐guide’, before the guide was released between March and May 2023. Prenatal care was routine during this period. The guide (see QR code below to view a pdf version of the guide) was then released, and a washout period was observed. The second group of patients, ‘post‐guide’, was surveyed between October–December 2023. Practice changes that impacted the ‘post‐guide’ cohort included medical assistants disseminating the third trimester guide to patients who were in the third trimester of pregnancy (ideally around 28−32 weeks). The surveys included 5‐point Likert Scales assessing patients' reported adequacy of counseling regarding a variety of prenatal topics (scale 1−5, 1 = very inadequate, 5 = very adequate), level of anxiety about the unknown (scale 1−5, 1 = not stressed at all, 5 = very stressed), as well as satisfaction with overall third trimester and delivery counseling (scale 1−5, 1 = very dissatisfied, 5 = very satisfied). The survey also inquired about the desire for additional education, participation in hospital in hospital tours and/or childbirth classes, and opinions on the third trimester guide. We performed *t*‐tests and chi‐squared analyses for comparison between the groups. We used an intention to treat analysis for the groups as likelihood of receiving the guide was variable in post‐guide group. A secondary analysis was performed comparing participants who reported receiving the guide with those who did not.


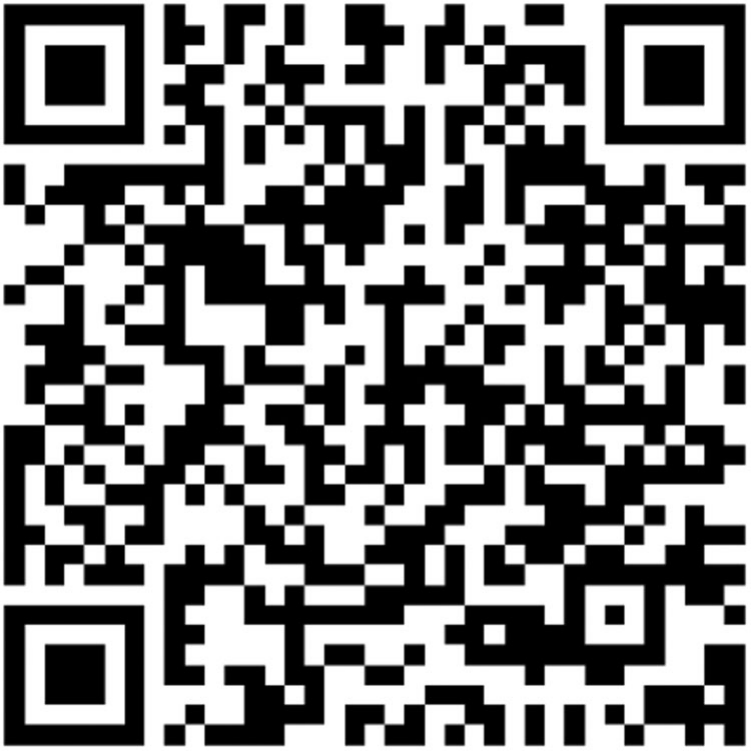


## 3 Results

One hundred percent (99/99) of patients approached agreed to participate. We enrolled 49 participants in the pre‐guide cohort and 50 in the post‐guide cohort. Table 1 displays pre‐ versus post‐guide cohorts' social demographics as well as information about the location/type of care that received. We found the cohorts were similar in social demographics other than a lower education level in the post‐guide cohort (*p* = 0.01, Table 1).

**Table 1:** Participant sociodemographics comparing pre‐ and post‐pregnancy educational guide introduction cohorts at an urban academic hospital in 2023.

	Pre‐guide	Post‐guide	
	*N* = 49	*N* = 50	
	*N* (%)	*N* (%)	*p* value
Prior number of children			
None	28 (57)	19 (38)	
One	10 (20)	12 (24)	0.13
Two or more	11 (23)	19 (38)	
Parity			
Nulliparous	28 (57)	19 (38)	0.06
Multiparous	21 (43)	31 (62)	
Education level			
Some high school or high school grad	8 (16)	21 (42)	0.01
Some college or college grad	19 (39)	17 (34)	
Post graduate education	22 (45)	12 (24)	
Location of care			
Resident clinic	12 (25)	21 (42)	0.07
Faculty practice	37 (76)	28 (58)	
Type of provider			
Midwife	11 (22)	13 (26)	0.68
Physician	38 (78)	37 (74)	

Table 2 shows the pre vs. post cohorts' perception of adequacy of counseling on a variety of the topics explained in the third trimester guide. There were generally no significant differences in the pre‐guide cohort vs. the post‐guide cohort (Table 2). Patients in both groups felt that prenatal counseling was most adequate in ‘changes in your body during pregnancy’. Both groups' average rating of adequacy in counseling across all the topics was 4 out of 5. We found no difference between the groups on patient reported stress level during pregnancy, their need to seek additional educational information or reporting the need for increased prenatal counseling or education classes. Interestingly, fewer in the post‐guide group reported being offered childbirth education classes (44% vs. 63%, *p* = 0.06). There was no statistically significant difference in whether groups were offered tours of hospital facilities (37% vs. 41%, *p* = 0.43). Both groups reported similar satisfaction with childbirth education classes (2.8 vs. 2.5 out of 5, *p* = 0.24) and tours of hospital facilities (2.4 vs. 2.1 out of 5, *p* = 0.19).

**Table 2:** Patient report of counseling adequacy of prenatal topics comparing pre‐ and post‐ pregnancy educational guide introduction cohorts at an urban academic hospital in 2023 (scale of 1−5 with 1 being ‘very inadequate’ and 5 being ‘very adequate’).

	Pre‐guide	Post‐guide	
	*N* = 49	*N* = 50	
Topic	Mean (SD)	Mean (SD)	*p* value
Tests performed during pregnancy	4.4 (1.1)	4.2 (1.1)	0.56
Relevant and interesting information on how to take care of yourself	4.1 (1.3)	4.1 (1.1)	0.87
Changes in your body during pregnancy	4.8 (5.7)	4.5 (4.1)	0.42
How your baby should be moving during pregnancy	4.3 (1.1)	4.4 (1.0)	0.58
Stress level during pregnancy	2.6 (1.4)	2.7 (1.2)	0.69
Tests performed during pregnancy	4.4 (1.1)	4.2 (1.1)	0.56
Pre‐eclampsia symptoms	3.7 (1.4)	3.7 (1.5)	0.90
How to prepare for your hospital delivery (i.e., what to pack)	3.4 (1.4)	3.6 (1.3)	0.36
Pain management during childbirth	3.5 (1.5)	3.6 (1.4)	0.65
Childbirth: symptoms and process	3.9 (1.3)	3.7 (1.4)	0.41
Breastfeeding	3.6 (1.5)	3.8 (1.3)	0.55
Birth control options	4.1 (1.3)	4.3 (1.1)	0.56
Average across all counseling topics	4.0 (1.1)	4.0 (3.7)	0.92

Overall satisfaction with counseling about the third trimester and delivery topics was high and similar between the pre and post‐guide cohorts (4.0 SD 1.0 vs. 4.0 SD 1.1, *p* = 0.92). Satisfaction with counseling was not significantly associated with the location of care, the participant's education level, the type of provider from whom they received care, or the participant's parity.

There was no significant difference in the percentage of patients in each group who reported needing to search for additional information (59% vs. 49%, *p* = 0.31) and wanting more counseling (47% vs. 38%, *p* = 0.40).

Of note, only 28/49 (57%) of the post‐guide cohort reported that they had received the guide. Patients were shown a physical copy of the guide if they were unsure if they had received it prior. Of those that received the guide, 78% (21/27) shared it with their partners.

As a secondary analysis we compared those in the post‐guide cohort who received the guide versus those who did not and found no significant difference in their satisfaction with third trimester and delivery counseling. When comparing adequacy of counseling among prenatal topics, the only topic where patients who received the guide reported a statistically significant difference in counseling adequacy was in ‘pain management in childbirth’ (4.1 SD 1.1 vs. 3.4 SD 1.5, *p* = 0.001). Importantly, of those participants who received the guide, 75% felt that it reduced their anxiety and stress about the unknown and 100% would recommend it to others. Of those that received the guide, the average rating on how they perceived the length of the guide (one being too short, three being just right, five being too long) was 2.7 95% CI (2.5−2.9), indicating the 15‐page guide was on the shorter side of their preference.

## 4 Discussion

We did not find that introduction of an informational guide about the third trimester of pregnancy significantly affected patient satisfaction with third trimester and delivery counseling or with their reported adequacy of counseling of pregnancy topics. Almost half of the participants who were supposed to receive the guide did not, indicating a need to improve office workflows for distributing educational content. However, of our participants who received the guide, they reported both that it reduced their anxiety and that they would recommend it to others. These findings are in alignment with prior studies that show high levels of satisfaction with educational materials themselves and that they play a role in relieving anxiety [7, 8]. Furthermore, a large proportion of patients who received the guide shared it with their partners, which we hope can benefit partners who might not be able to be present at prenatal appointments as well as serve as way to spark discussion about prenatal topics. Based on this positive response to the third trimester guide itself, we suggest written educational materials as a cost‐efficient and adequate adjunct to information received during prenatal appointments, in‐person childbirth classes, and hospital tours, especially as the latter two were not commonly utilized by our study population. We hope that these targeted educational interventions can be a valuable component of health‐care spending policy and spending, contributing to better‐informed and more confident patients.

Some strengths of this study include that we were able to surpass the desired sample size based on the sample size calculations as mentioned above. Furthermore, out of all patients approached for participation, 100% of the patients agreed to complete the survey and did so. This suggests that the survey itself was an appropriate length and accessible to our patients. Our study is novel in our third trimester pregnancy guide intervention and by sharing our guide in this publication we hope to improve information that may be distributed at other health care institutions.

Some limitations to this study include low rate of reported distribution of the third trimester guide, which blunted our ability to analyze our primary research question. However, this reflects a common real‐world challenge with QI initiatives. Additionally, there was a significant difference in reported education level between on our two cohorts, which may have been a confounding variable in our research. Finally, the fact that the educational guide was offered only in English limited our study of patients who do not speak/read English, which is a sizeable proportion of the patient population in our hospital.

Future directions for research may involve understanding how the format of educational materials (physical vs. electronic) affect patient satisfaction with prenatal counseling and analyzing if/how patient satisfaction changes with regard to materials geared toward earlier trimesters of pregnancy. We also would have been interested in evaluating patient understanding of topics after receiving this educational guide.

## 5 Conclusions

We did not find that introduction of a new informational guide about the third trimester of pregnancy significantly affected patient satisfaction with counseling about the third trimester and delivery. Almost half of the participants who were supposed to receive the guide did not, indicating a need to improve office workflows for distributing educational content. However, our findings showed that patients viewed the educational guide positively; participants who received the guide reported satisfaction with the guide itself, that it lessened their anxiety, and that they would recommend it to others.

## Conflicts of Interest

The authors declare no conflicts of interest.

## Data Availability

The data that support the findings of this study are available from the corresponding author upon reasonable request.
